# Efficacy and safety of robotic spine surgery: systematic review and meta-analysis

**DOI:** 10.1186/s10195-022-00669-0

**Published:** 2022-10-15

**Authors:** Setefilla Luengo-Matos, Luis María Sánchez-Gómez, Ana Isabel Hijas-Gómez, Esther Elena García-Carpintero, Rafael Ballesteros-Massó, Mar Polo-deSantos

**Affiliations:** 1 Health Technology Assessment Agency (Agencia de Evaluación de Tecnologías Sanitarias, AETS), Carlos III Institute of Health, Madrid, Spain; 2 Research Network on Chronicity, Primary Care and Health Promotion (RICAPPS), Madrid, Spain; 3 Servicio de Traumatología, Hospital Quirónsalud Sur, Alcorcón, Spain; 4 Consejo Superior de Investigaciones Científicas, Madrid, Spain

**Keywords:** Spine, Robotic surgery, Pedicle, Systematic review

## Abstract

**Background:**

Robotic surgery (RS) may offer benefits compared with freehand/conventional surgery (FS) in the treatment of patients with spinal disease. The aim of this study was to evaluate the efficacy and safety of RS versus FS in spinal fusion.

**Methods:**

A systematic review and meta-analysis was performed. Data analysis and risk of bias assessment were analysed using REVMAN V5.3.

**Results:**

We found 11 randomised clinical trials involving 817 patients (FS: 408, RS: 409). The main diagnosis was degenerative spine disease. SpineAssist, Renaissance (Mazor Robotics), Tianji Robot and TiRobot robots (TINAVI Medical Technologies) were used. Pedicle screw placement within the safety zone (grades A + B according to the Gertzbein and Robbins scale) ranged from 93% to 100% in FS versus 85–100% in RS (relative risk 1.01, 95% CI  1.00–1.03, *p* = 0.14). Regarding intervention time, the meta-analysis showed a mean difference (MD) of 6.45 min (95% CI  −13.59 to 26.49, *p* = 0.53). Mean hospital stay was MD of −0.36 days (95% CI  −1.03 to 0.31, *p* = 0.30) with no differences between groups. Contradictory results were found regarding fluoroscopy time, although there seems to be a lower radiation dose in RS versus FS (*p* < 0.05). Regarding safety, the studies included surgical revision frequency.

**Conclusions:**

No conclusive results were found suggesting that there are benefits in using RS over FS for spinal fusion. Further research with adequate patient selection, robot type and quality-of-life variables is needed.

*Level of evidence*: level 1.

**Supplementary Information:**

The online version contains supplementary material available at 10.1186/s10195-022-00669-0.

## Introduction

Spinal diseases are a major public health problem. They involve different processes of various aetiologies, although the most frequent are degenerative, closely linked to the ageing of the population [1]. The most serious cases are associated with an increase in chronicity, deterioration in quality of life and reduction in the patient’s autonomy [2]. Their cost to the health system is high and has been rising in recent years [3].

The treatment of spinal diseases usually begins with a conservative approach aimed at the management of symptoms [1]. However, it is sometimes not effective, and these patients are candidates for surgical treatment [4]. The most common procedure is arthrodesis or spinal fusion. It consists of creating a bone bridge between two or more adjacent vertebrae by implanting bone tissue grafts or bone substitutes between the vertebrae to be fused [5]. The most commonly used instruments for fixation are pedicle screws and bars which, by stabilising the vertebral segments, facilitate the formation of bone tissue between these vertebral segments forming a solid mass [1].

The traditional way of placing pedicle screws is by means of the “freehand technique”, conventional or manual (FS). The technique uses local anatomical references to identify the entrance to the pedicles and achieves good accuracy in screw placement [6]. On occasion, re-intervention is necessary owing to complications arising from malposition of the screws [7]. To improve the accuracy of screw insertion, new surgical assistance devices have been incorporated such as fluoroscopy, navigation systems with intra-operative 3D fluoroscopy or, more recently, robots [6, 8, 9].

In general, robot-guided surgical procedures, prior to the operation, consist of of a computed tomography (CT) scan that allows for three-dimensional reconstruction, vertebra by vertebra, to assist in planning. The information from the CT scan is transferred to the robot in the operating room, which is fixed to the patient’s spine, allowing anatomical relationships and precision to be maintained at all times. It is the robot, moving along the vertebrae, that guides the approach for accurate and reliable implant placement [10].

The fundamental measure of efficacy to assess the outcome of the surgical procedure is the precision of the placement of the pedicle screws. The most commonly used scale is the Gertzbein and Robbins scale, which classifies screw position into five grades, where the highest precision corresponds to grade A and the lowest precision corresponds to grade E [11].

In addition to the possible benefits of robotic surgery (RS) in terms of precision in the placement of pedicle screws, possible positive effects have been described in relation to surgical time, hospital stay or complications [12]. However, some studies show non-conclusive results in favour of RS, and it is not clear whether the use of the robot would justify its incorporation into clinical practice, given its high acquisition and maintenance costs [3, 13].

The aim of this systematic review is to analyse the efficacy and safety of RS treatment versus conventional FS in the placement of screws in patients undergoing spinal surgery.

## Methods

We performed a systematic review in accordance with PRISMA guidelines [14], with the methods of the analyses and inclusion criteria being specified in advance and documented in a protocol. We searched MedLine, EMBASE, Cochrane Library and other databases of health technology assessment agencies. The search period was until April 2019, and was updated until April 2021. A manual review of the bibliographic references of the documents found was also carried out. The search strategy did not include restrictions on study size.

The selection of relevant studies was based on the Population–Intervention–Comparator–Outcome-Study Design (PICOS) criteria (Table 1). Studies in English, French and Spanish were included. Studies that failed to meet the PICOS criteria or provide assessable data related to the selected outcome measures were excluded. Similarly, we excluded studies that were duplicated or outdated by subsequent studies by the same institution.

**Table 1:** Inclusion criteria according to the PICOS scheme

Population	Patients of any age and sex with any pathology of the spine
Intervention	Robot-assisted surgery for the placement of pedicle screws in spinal operations
Comparator	Any other type of surgery for the placement of pedicle screws in spine surgery
Outcomes	Any measure related to the efficacy and safety of the use of the robot. Studies assessing economic, organisational, ethical, legal or implementation aspects of the technology were also included
Study design	Randomised controlled trials (RCTs), SRs and/or meta-analyses, HTA reports, Clinical Practice Guidelines

The identification, selection, review, data extraction and assessment of the evidence of studies was carried out by two independent reviewers, with any discrepancies being resolved by consensus, and a third reviewer being consulted in case of disagreement. Tables were prepared detailing the studies included and excluded in the review, justifying the cause of exclusion (available to the reader).

Meta-analyses were carried out in relation to the accuracy of the placement of the pedicle screws, the duration of the intervention, and the hospital stay in FS and RS, using the random effects model to take into account the heterogeneity among the studies. The degree of heterogeneity was assessed using graphic and statistical methods (*χ*^2^ statistic and *I*^2^ inconsistency index). Relative risk (RR) and mean difference (MD) were used as relative measures of effect and presented graphically in the corresponding forest plots, with their 95% CIs.

A funnel plot was used to assess the presence of publication bias, interpreting a symmetrical inverted V-shaped graph as a demonstration that there is probably no publication bias. Data analysis was carried out using REVMAN V5.3 [15], which uses the Cochrane risk of bias assessment tool for RCTs [16]. We used the GRADE methodology to assess the quality of the evidence [17]. Similarly, an internal quality assessment was performed using the checklist developed within the framework of the Spanish Network of Health Technologies Assessments Agencies (RedETS), and an external review by a specialist in Orthopaedic Surgery.

## Results

**Fig. 1:**
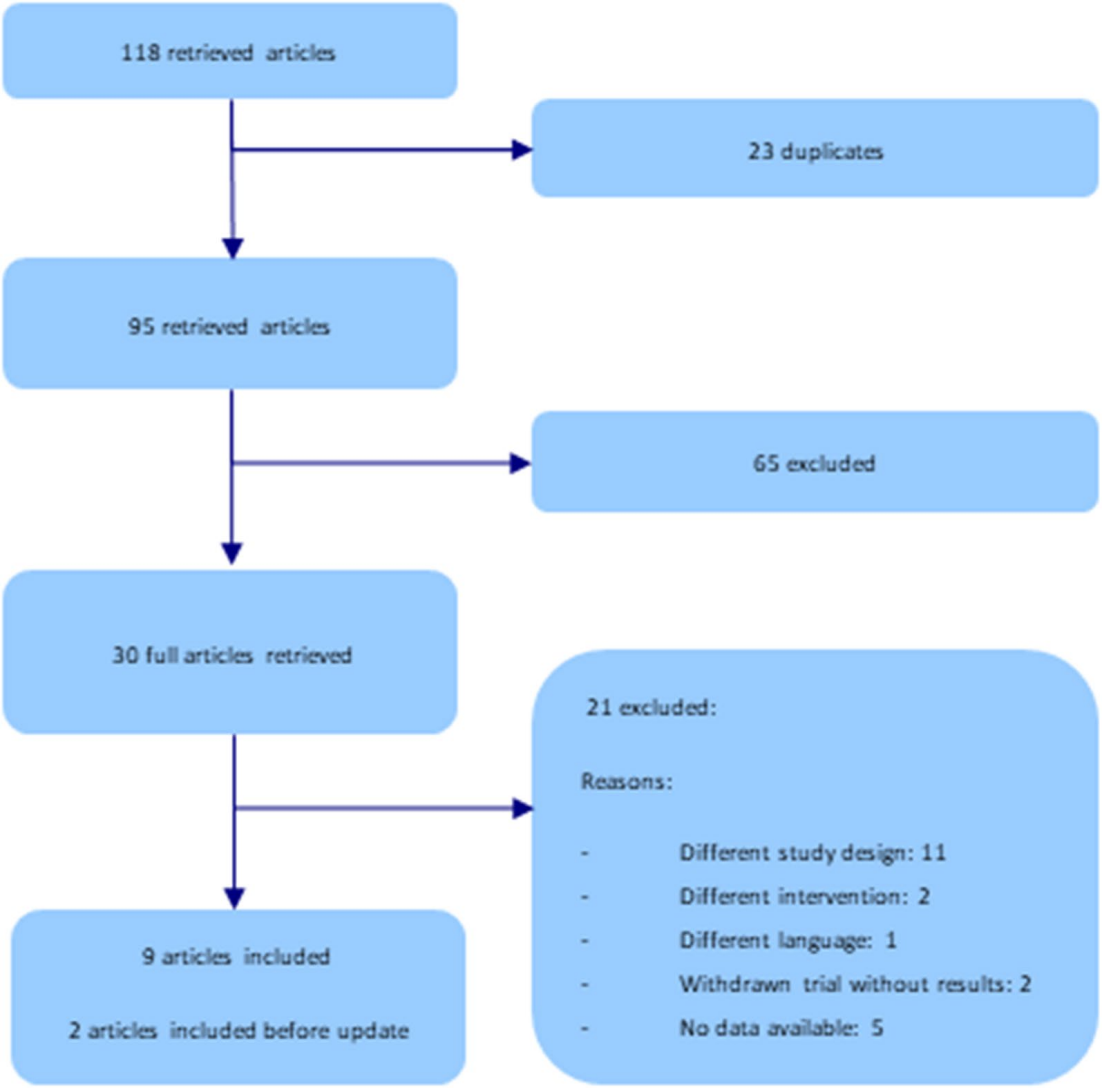
Study flow diagram

Our electronic search identified 118 articles. After screening the title/abstracts, we retrieved the full text of 30 references, of which 21 were excluded. We included nine studies that analysed spinal arthrodesis with FS versus RS [13, 18–25]. Two of the included studies correspond to the same trial [22, 24]; the second study [24] provided additional data on the quality of life of patients 1 year after the intervention. The update of the literature search identified two further studies [26, 27] (Fig. 1 Study flow diagram). Trials were published between 2013 and 2020. The trials were performed in Germany [18, 19], China [13, 20, 23, 25–27] and Korea [21, 22, 24]. One trial declared that they received industry help (equipment loan) [22, 24], while the other trials received no funding.

### Participants

A total of 817 participants were analysed, 408 undergoing FS and 409 with RS. The mean age ranged from 49 to 67.9 years (FS: 49.5–67.9 years; RS: 49–67.6 years). In five studies, the percentage of female patients operated on, in both FS and RS, was higher (FS: 51.3–73.3%; RS: 52.2–70.0%) [13, 18, 21, 25, 27], while in four other studies no differences were observed or the percentage was slightly lower [19, 20, 22, 23]. The main diagnosis was degenerative spine disease in most studies, and two studies also included traumatic pathology [13, 23] (Table 2).

**Table 2:** Summary of patient characteristics

	Patients (*N*)	Age (years) (mean ± SD)	Sex *n* (%)		BMI (kg/m^2^) (mean ± SD)	Diagnosis	Symptom length (months) (mean ± SD)
			Women	Men			
Ringel [18]							
Freehand	30	67*	18 (66.0)	12 (40.0)	28*	Indication for lumbosacral stabilisation	–
Robot	30	68*	16 (53.3)	14 (46.4)	26*		
Roser [19]							
Freehand	10	–	–	–		Degenerative lumbar instability	–
Robot	18	–	–	–			
Hyun [21]							
Freehand	30	66.8 ± 8.9	22 (73.3)	8 (26.7)	25.8 ± 3.3	Degenerative lumbar disorder	–
Robot	30	66.5 ± 8.1	21 (70.0)	9 (30.0)	24.7 ± 2.6		
Kim [22]							
Freehand	41	66.0 ± 8.6	19 (46.3)	22 (53.7)	25.3	Lumbar spinal stenosis	13.1 ± 8.2
Robot	37	65.4 ± 10.4	18 (48.6)	19 (51.4)	25.9		12.5 ± 9.3
Tian [20]							
Freehand	17	–	–	–	–	–	–
Robot	23	–	–	–	–		
Wang [23]							
Freehand	15	43*	7 (46.7)	8 (53.3)	–	Polytrauma	–
Robot	15	36*	5 (33.3)	10 (66.7)	–		
Feng [25]							
Freehand	40	67.9 ± 7.3	27 (67.5)	13 (32.5)	25.6 ± 3.5	Degenerative disk disease: 19 Degenerative spondylolisthesis: 12 Spondilolytic listhesis: 5 Degenerative scoliosis: 4	–
Robot	40	67.6 ± 6.5	28 (70.0)	12 (30.0)	25.0 ± 4.5	Degenerative disk disease: 20 Degenerative spondylolisthesis: 10 Spondilolytic listhesis: 7 Degenerative scoliosis: 3	
Han [13]							
Freehand	119	56.1 ± 13.4	61 (51.3)	58 (48.7)	24.9 ± 2.9	Degenerative pathology: 84 Traumatic pathology: 35	–
Robot	115	54.6 ± 11.3	60 (52.2)	55 (47.8)	25.7 ± 4.1	Degenerative pathology: 74 Traumatic pathology: 41	
Fan [26]							
Freehand	66	49.5 (39,59)	27 (40.9)	39(59.1)	24.47 ± 3.94	–	
Robot	61	49 (34.5,57.5)	18 (29.5)	43(70.5)	23.65 ± 4.10	–	
Feng [27]							
Freehand	40	64.22 ± 6.19	25(62.5)	15(37.5)	–	Lumbar spinal stenosis: 21 Degenerative spondylolisthesis: 14 Lumbar instability: 5	
Robot	40	63.45 ± 4.56	24(60)	16(40)	–	Lumbar spinal stenosis: 19 Degenerative spondylolisthesis: 18 Lumbar instability: 3	

*BMI* body mass index, *SD* standard deviation

^*^Median

### Surgical characteristics

In both FS and RS, the most common surgical approach was the posterior approach [13, 21, 22, 25]. The total number of screws placed, including pedicle screws and other cervical screws, ranged from 22 to 584 in FS and from 23 to 532 in RS. Only one study included the average number of pedicle screws used per operation with a mean of 4.7 screws in FS versus 4.3 in RS [21]. Another study specified the diameter of the screws (6.5 and 5.5 mm) [22]. The single segment (two adjacent vertebrae) was the most common arthrodesis in both FS and RS [19, 21–23]. The most frequent level of arthrodesis was lumbar [13, 18, 19, 21, 22, 25, 27]. The robots used were the SpineAssist Surgical Guidance Robot [18, 19] and the Renaissance Surgical Guidance Robot [21, 22] from Mazor Robotics; the TiRoboT [13, 20, 23, 25, 27] and Tianji Robot, only in the cervical region, from TINAVI Medical Technologies [26] (Table 3).

**Table 3:** Surgery characteristics

	Approach	Decompression	Total pedicle screws (*n*)	Fusion level (percentage of patients)		Vertebral level	Robot
				One segment *n* (%)	Two segment *n* (%)		
Ringel [18]							
Freehand	–	If needed	152	14 (46, 7)^a^	16 (53, 3)^a^	L2: 8 L3: 30 L4: 52 L5: 52 S1: 10	–
Robot		If needed	146	17 (56, 7)^a^	13 (43, 3)^a^	L2: 8 L3: 24 L4: 50 L5: 48 S1: 16	SpineAssist
Roser [19]							
Freehand	Posterolateral	Yes	40	10 (100)^a^	–	Lumbar	–
Robot	–	–	72	18 (100)^a^	–	Lumbar	SpineAssist
Hyun [21]							
Freehand	Posterior	–	140	20 (66, 7)^a^	10 (33, 3)^a^	Lumbar	–
Robot	Posterior	If needed	130	25 (83, 3)^a^	5 (16, 7)^a^	Lumbar	Renaissance
Kim [22]							
Freehand	Posterior	Yes	172	37 (90, 2)^a^	4 (9, 8)^a^	L2–3: 2	–
Robot	Posterior	Yes	158	32 (86, 5)^a^	5 (13, 5)^a^	L2–3: 3	Renaissance
Tian [20]							
Freehand	–	–	88	–	–	–	–
Robot	–	–	102	–	–	–	TiRobot
Wang [23]							
Freehand	–	–	22	22 (100)^b^	0	S1: 13 S2: 9	–
Robot	–	–	23	19 (82, 6)^b^	4 (17, 4)^b^	S1: 13 S2: 10	TiRobot
Feng [25]							
Freehand	Posterior	If needed	225	–	–	L2: 18 L3: 49 L4: 78 L5: 80	–
Robot	Posterior	If needed	202	–	–	L2: 18 L3: 48 L4: 80 L5: 64	TiRobot
Han [13]							
Freehand	Posterior	If needed	584	–	–	Thoracic and lumbar	–
Robot	Posterior	–	532	–	–	Thoracic and lumbar	TiRobot
Fan [26]							
Freehand	–	If needed	204^c^	–	–	Cervical	Tianji Robot
Robot	–	If needed	186^c^	–	–	Cervical	
Feng [27]							
Freehand	Posterior	–	174	–	–	Lumbar	TiRobot
Robot	–	–	170	–	–	Lumbar	

^a^Number of patients

^b^Number of pedicle screws

^c^These data include all types of cervical screw: number of lateral mass screws, 117 (69 for freehand and 48 for robot procedure); number of odontoid screws, 38 (21 for freehand and 17 for robot); number of Magerl screws, 60 (25 for freehand and 35 for robot); number of pedicle screws, 175 (89 for freehand and 86 for robot)

### Risk of bias in included studies

**Fig. 2:**
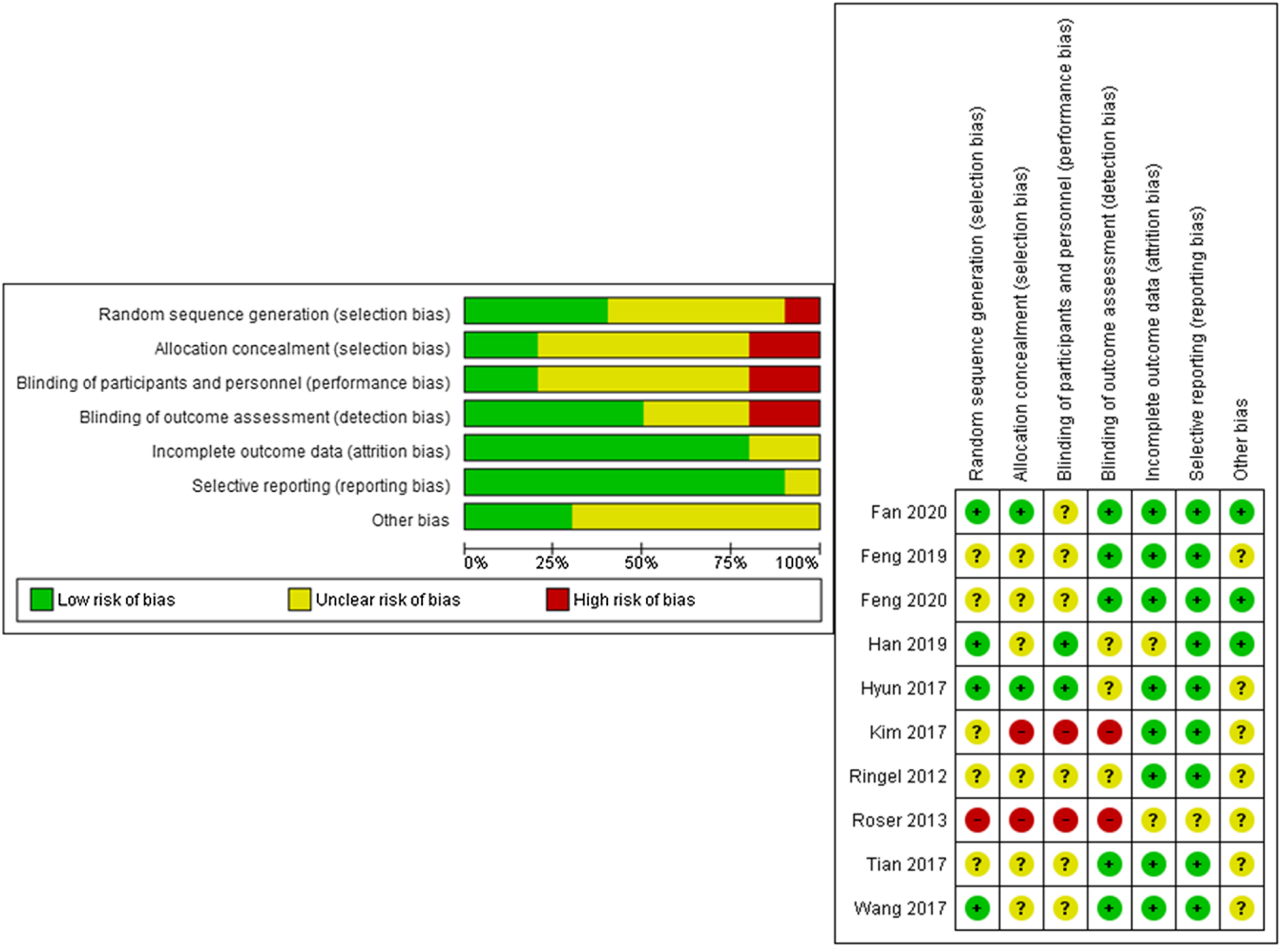
Risk of bias in included studies

Random sequence generation, allocation concealment, blinding of participants and personnel, blinding of outcome assessment and others were judged as at an unclear/high risk of bias in most of studies. Incomplete outcome data and selective reporting were judged as at a low risk of reporting bias (Fig. 2 Risk of bias included studies). No publication bias was identified.

### Certainly of evidence

The certainty of the evidence has been rated as low or very low owing to the high risk of bias observed in the studies and the high heterogeneity observed with *I*^2^ values ranging between 34% and 93% (Table 4). For some outcomes, the quality of evidence has been downgraded for imprecision due to the small size of the samples analysed.

**Table 4:** Main findings of studies included in meta-analysis

	Patient (studies)	Surgical events (events/total)		Risk ratio/mean difference (95% CI)	Overall certainty of evidence
		Freehand	Robot		
Accuracy of pedicle screw placement^a^					
Grade A (maximum accuracy)	3477 (9 RCTs)	1515/1779	1566/1698	RR 1.06 (1.01–1.11) *p* = 0.02 (*I*^2^ = 87%)	Very low
Grades A + B, safety zone	3477 (9 RCTs)	1706/1779	1665/1698	RR 1.06 (1.01–1.11) *p* = 0.14 (*I*^2^ = 81%)	Very low
Proximal facet violation	1716 (3 RCTs)	26/896	0/820	RR 0.07 (0.01–0.40) *p* = 0.003 (*I*^2^ = 0%	Low
Intra-operative blood loss (ml)	394 (3 RCTs)	199	195	MD −68.12 (−109.24 to 27.01) *p* = 0.001 (*I*^2^ = 34)%	Very low
Radiation dose (standard mean difference)^b^	402 (4 RCTs)	203	199	MD −1.31 (−2.02 to −0.60) *p* = 0.0003 (*I*^2^ = 87%)	Very low
Fluoroscopic time (min)	262 (2 RCTS)	50	58	MD −3.00 (−28.01 to 22.00) *p* = 0.81 (*I*^2^ = 93%)	Very low
Total screw placement time (min)	108 (2 RCTs)	50	58	MD 0.84 (−10.93 to 12.61) *p* = 0.89 (*I*^2^ = 89%)	Very low
Operating time (min)	492 (5 RCTs)	247	245	MD 6.45 (−13.59 to 26.49) *p* = 0.53 (*I*^2^ = 74%)	Low
Length of hospital stay (days)	374 (3 RCTs)	189	185	MD −0.36 (−1.03 to 0.31) *p* = 0.30 (*I*^2^ = 62%)	Very low

*MD* mean difference, *RR* risk ratio

^a^Assessed with: Gertzbein and Robbins scale

^b^Measurements were made in different units: µSv, mSv and mGy

### Effects of intervention

**Fig. 3:**
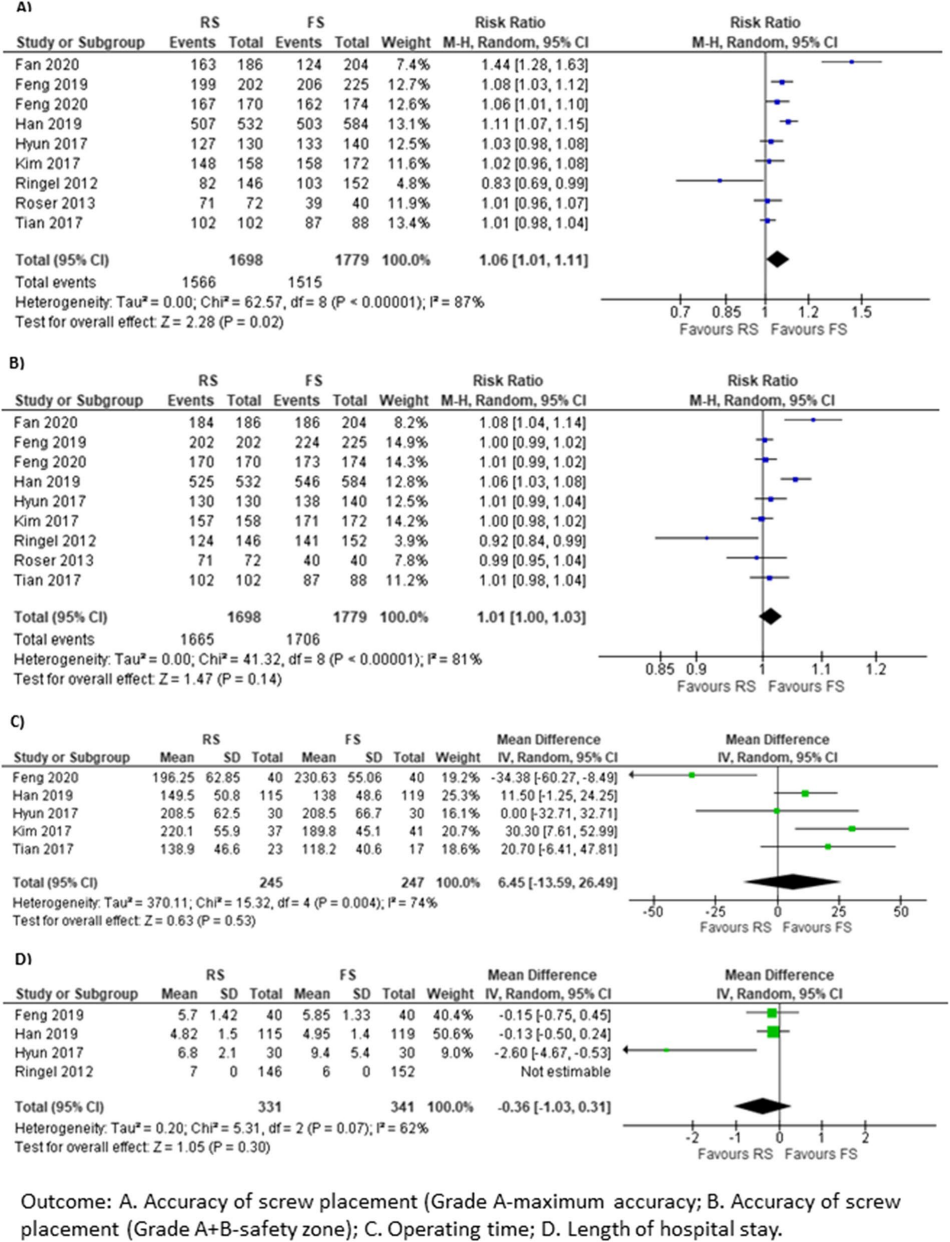
Results of meta-analysis

The main efficacy and operation-related outcomes are listed in Table 4; the other outcomes and the quality of evidence can be found in the supplementary material (Additional file 1). Precision of pedicle screw placement was evaluated using the Gertzbein and Robbins scale [11] in most of the studies [13, 18–22, 25–27] and according to the criteria of Gras et al. [28] in one study [23]. According to the Gertzbein and Robbins scale, the maximum precision (grade A) was obtained in 68.0–98.3% of the screws placed by FS and in 56.0–98.6% by RS. The meta-analysis of nine studies [13, 18–22, 25–27] did not show superiority of RS over FS (RR 1.06, 95% CI 1.01–1.07, *p* = 0.02, *I*^2^ = 87%). Accuracy of pedicle screw placement within the safety zone (grades A + B) was 93.0–100% in FS and 85.0–100% in RS, with no statistically significant difference between RS and FS (RR 1.01, 95% CI 1.00–1.03, *p* = 0.14). Only Ringel et al. [18] described favourable results for RS versus FS. There was marked heterogeneity among these nine studies, and a randomised method was used (*I*^2^ = 81%; *p* < 0.00001) (Fig. 3 Results of meta-analysis). According to the criteria of Gras et al. [28], the accuracy in screw placement was “excellent” in 72.7% of the cases with FS and in 100% with RS [23].

Only two studies evaluated the screw mean distance from the proximal facet, ranging from 2.7 ± 1.6 mm to 4.6 ± 0.6 mm in FS and 5.2 ± 2.1 to 5.8 ± 1.7 mm in RS (*p* < 0.01) [18, 19]. Four studies reported that the most common deviation was lateral (70.6% in FS and 34.2% in RS) [18, 20–22]. Additionally, four studies described intra-operative blood loss as a secondary outcome, with a variability between 254.7 and 165 ml for RS and 356.2 and 217 ml for FS [13, 25–27].

The use of RS was characterised by a lower radiation dose required in the intervention compared with FS, according to the findings of four studies [13, 19, 21, 25].

Other secondary outcomes included intervention-related times. Among them, the mean total fluoroscopy time did not differ significantly between FS and RS in the two studies that analysed this outcome [13, 19]. The mean operating time ranged from 118.2 to 230.6 min for FS and from 138.9 to 208.5 min for RS (MD 6.45 min, 95% CI  −13.59 to 26.49, *p* = 0.53), with a high heterogeneity among the studies (*I*^2^ = 74%). Pedicle screw placement time ranged from 27.8 ± 87.0 to 32.3 ± 10.5 min in the FS group versus 27.6 ± 8.6 min to 35.2 ± 11.3 min in the RS group [19, 25]. The mean and median planning time required in RS was 20 ± 5.3 min [19] and 7.8–24 min, respectively [18, 23].

According to the results of four studies [13, 18, 21, 25], the average time spent in hospital ranged from 5.0 to 9.4 days in FS and from 4.8 to 7.0 days in RS (MD −0.36 days, 95% CI  −1.03–0.31, *p* = 0.30). There was moderate heterogeneity between studies (*I*^2^ = 62%; *p* = 0.07), with only one study showing statistically significant differences with a shorter time spent in hospital in the RS group [21].

Four studies incorporated clinical results after a follow-up period ranging from 6.0 to 16.3 months on average [21, 22, 24, 27]. Improvements in both low back and lower limb pain measured with the EVA scale, quality of life measured with the SF-36, and disability measured with the Oswestry Disability Index (ODI) were described in both the FS and the RS groups, with significant differences between RS and FS in the ODI index alone in one study [24].

### Security

Eight studies collected information on the need for surgical revision to assess screw placement [13, 18, 20–23, 25, 26]. The number of surgical revisions ranged from 0 to 2 in FS and from 0 to 10 in RS; one of the studies described a significantly lower number of surgical revisions in the FS group than in the RS group (1 revision in 152 screws versus 10 in 146 screws; *p* < 0.05) [18]. No study reported on technical failures of the procedure or cases of death. Two studies described other adverse events, such as wound infection [26, 27], although no difference in infections rates between groups was observed. Other adverse events were three cases of post-operative cerebrospinal fluid fistula headache, one case of vertebral artery injury without symptom and one case of weakness in the left hip flexor in the FS group [26, 27].

### Other outcomes of interest

No studies on cost or cost-effectiveness were found. One study described that the price of the Renaissance system, including hardware and installation cost, was $550,000 in 2018, not including disposables and implants (about $1500 per case); in addition, the system’s maintenance costs should be considered [2]. No studies assessed organisational, ethical, legal or implementation aspects of the technology.

## Discussion

The present study aims to determine the efficacy and safety of RS versus FS in spinal fusion. Eleven clinical trials that respond to the objective of our research were analysed. We found that in both FS and RS the socio-demographic characteristics of the patients were similar. The most common surgical approach was posterior, the most frequent arthrodesis was monosegmentary and the most frequent location was at the lumbar level. We have not found sufficient information on whether the cases operated on with RS were minimally invasive or open surgery. The robot seemed to benefit minimally invasive surgery by guiding the surgeon to the precise location without the need for anatomical visualisation [21, 29]. In cases in which open surgery with visualisation of the surgical field is required, the robot would provide fewer advantages [29].

The robots used are essentially two: first and second generation from Mazor Robotics ([18, 19, 21, 22] and TiRobot [13, 20, 23, 25, 27]. However, there are other different types of robots on the market, and the technological development of these devices is evolving rapidly. It is expected that the new generations of robots are designed to have fewer limitations and greater ease of use [3, 19, 29]. Selecting the type of robot is important since the results can vary according to the type of robot used or the system of navigation chosen [3].

Precision in the placement of the pedicle screws is described as the fundamental goal to achieve in spinal fusion surgery [8]. To measure precision, the Gertzbein and Robbins scale is used [11], although the studies did not detail how the information was collected. Maximum precision (grade A) or the placement of pedicle screws within the safety zone (grades A + B) was achieved in a high percentage of cases in both FS and RS. The results of the meta-analysis show a result slightly in favour of FS. However, the studies show great heterogeneity, so these results should be treated with caution. Some problems concerning lack of precision in RS were attributed to the system of fixation of the robot to the patient’s spine [18]. The literature has shown contradictory results regarding accuracy of RS. There are studies that observe a clear superiority of RS over FS [30, 31], while others observe no differences between groups [32, 33]. However, a high heterogeneity is also noted between studies.

In addition to accuracy, the studies analysed other variables related to screw placement, such as distance of the screw to the articular facet, screw deflection at the entry point and at the exit point, and invasion of the articular surface. The information collected in the studies was heterogeneous and limited, and it was not possible to adequately evaluate the results.

Other important outcomes included intra-operative blood loss, and radiation and fluoroscopy dose and time. Blood loss was higher in the FS group than in the RS group, although data were limited. This may be attributed to the fact that open FS usually involves greater soft tissue trauma with consequent blood loss, while RS is usually minimally invasive [21]. In cases where RS is performed openly, blood loss is also greater than in cases where a minimally invasive procedure is used [13].

In relation to radiation dose, understood as the cumulative fluoroscopy dose required for screw insertion [21], most studies showed that the dose was higher in the FS group than in the RS group, although the units of measurement used were different between studies. In the case of FS, the surgeon may continuously adjust the position of the screws during the procedure, resulting in a higher radiation exposure than occurs in minimally invasive SR. Owing to the associated radiation risk to the operating room staff and to the patient [13, 29], the lower radiation exposure is considered a relevant factor in favour of RS [21]. Regarding fluoroscopy time, the results are not clearly in favour of one or the other type of intervention, since the data are scarce. Nor was it possible to evaluate whether fluoroscopy time is decreased with repeated use of the robot [6].

We found that most of the studies analysed the relationship between the type of intervention and intervention time, and hospital stay. The meta-analysis did not show a significant difference in operating time between groups. For some authors, the screw placement time could be reduced with the help of the robot, and this represented a quarter of the total intervention time [12, 25]. The data found in the studies were insufficient to draw a conclusion in this regard. On the other hand, the mean hospital stay was similar in both intervention groups, despite the fact that the minimally invasive approach of SR would be expected to favour a shorter duration of hospitalisation [21].

Other efficacy outcomes, such as the ODI estimate, showed a superior improvement in the index after SR compared with after FS. On the other hand, the evaluation of efficacy indicators in relation to disability or quality of life was limited. We consider that, taking into account that arthrodesis essentially seeks to improve patients’ quality of life, the collection and analysis of these types of variables should be strengthened in future studies [4].

In relation to the safety of the technology, the studies reported the number of surgical revisions that had to be performed to assess the adequate placement of the screws, with similar results in both intervention groups, with no information on associated deaths. Only two studies reported on adverse events, which included mainly wound infections and post-operative cerebrospinal fluid fistula headache, without significant difference between groups. However, it would be necessary to establish a procedure for maintaining the sterility of the robots [26]. Although the robot may provide advantages, it would not replace the surgeon’s knowledge of the surgical anatomy and ability to handle unforeseen events during the operation [29].

In assessing the results of this study, it is relevant to point out the importance of the learning curve in SR. The number of interventions required for the proper use of the first generation of the Mazor robot was estimated at 25, although new generations of robots may require a shorter learning time [29]. The two studies included that evaluated the learning curve showed contradictory results [18, 21]. It is essential that interventions be performed by experienced professionals [6].

We should keep in mind that our study focuses on the results of spinal fusion with FS versus SR. However, there are other surgical assistance procedures that have shown good results in terms of accuracy and safety [8]. One of the studies included in our review incorporated navigator-guided surgery in addition to FS and SR in the comparative analysis [19]. The study, which analysed nine patients, found similar screw placement accuracy results in the three intervention groups. Additionally, a retrospective study comparing SR with new generations of robots versus navigator-guided surgery with 3D tomography revealed that both procedures are safe and accurate. However, the robot required shorter fluoroscopy time, shorter screw placement time and shorter hospital stay. The authors stated that the results should be verified in future studies [12].

From an economic point of view, we have not found any studies on the cost of the technology or the profitability of the procedure. However, the price of the robot is high, with a high acquisition and maintenance cost [3]. Some authors argue that it may be time and resource consuming [19], although others suggest that the introduction of the technology could be reasonable in first-world healthcare systems [2]. One way to improve the cost-effectiveness of the robot would be to increase its indications. In this sense, some types of robots such as the TiRobot can be used in different anatomical locations, both in open surgery and in minimally invasive surgery, which could provide advantages [13]. We found no information regarding other organisational, ethical, legal or implementation aspects. However, some of the outcome variables collected in relation to efficacy, such as time of surgery or radiation dose required, may be related to these aspects.

We would like to point out the limitations of this study. The results may change depending on the search strategy chosen and the inclusion and exclusion criteria considered. Several sources of heterogeneity were observed among the studies, including the main cause of diagnosis, the type and use of the robot, and the outcomes analysed. On the other hand, first-generation robots and second-generation robots, analysed by the included studies, did not have integrated navigation and independent instrument navigation. Recent spine robots have a fully integrated navigation platform, allowing for real-time instrument tracking and pedicle screw placement without guidewires [34]. The data collected varied across studies; sometimes, the data were scarce and sometimes the units of measurement were different, so it is not possible to properly assess these findings. In addition, the risk of bias was difficult to define in most of the studies. Bias assessment reported using funnel plots should be interpreted with caution, since the number of studies was not sufficient according to the recommendations (ten or more included studies). Nevertheless, a comprehensive and systematic search of multiple databases and information sources was performed to reduce the potential for publication bias.

It is important to emphasise that progress is currently being made in the development of robots, with the aim of improving existing limitations, facilitating their use and achieving maximum benefits in terms of precision and safety [29]. The use of robotic assistance in spinal interventions is particularly relevant, as precision is crucial and the device can be adapted to limited surgical access. In this regard, new generations of cervical spine robots have been specifically designed to enable percutaneous interventions in the area with promising results [19]. Only one included study analysed the efficacy and safety of cervical spine robots, showing outcomes superior to those of FS [26], with screw deviation < 1 mm, which is considered to be the optimal expected accuracy for a surgical navigation system [35]. On the other hand, the deviation observed in this study is lower than that observed in other studies [13, 20, 26]. Ideally, and contributing to improving its efficacy, the extension of the use of robotic assistance to other types of interventions, and not exclusively for the spine, should be considered. The aim would be to assist different procedures, providing a common benefit between different surgical disciplines [19].

## Conclusion

The present study found no significant differences between FS and RS with respect to the primary outcome, accuracy of pedicle screw placement. It was not possible to adequately assess the results of other variables related to screw placement, such as distance of screws to the articular facet, screw deviation or invasion of the articular surface, as data are still scarce and the method of data collection differed from one study to another. No clear results were found in favour of one or the other type of intervention in terms of safety, total operative time, pedicle screw placement time or hospital stay. Surgical intervention time was shorter in the FS group than in the RS group, although the data are limited and the results should be interpreted with caution. Information on cumulative fluoroscopy dose required for screw insertion and fluoroscopy time was equally scarce.

The studies showed heterogeneity in the patients operated on, in the type and use of the robot, and in the results evaluated, and are not free of possible biases.

It is essential to perform new studies with an adequate selection of patients, type of robot, and comparator, including additional clinical and quality-of-life variables.

## Supplementary Information

**Additional file 1.** Table GRADE-Summary of findings.

## Data Availability

Data available in supplementary material.
