# Supplementary material for: Efficacy and safety of robotic spine surgery: systematic review and meta-analysis
Source: J Orthop Traumatol. 2022 Oct 15;23:49. doi: 10.1186/s10195-022-00669-0 (PMC9569281; doi:10.1186/s10195-022-00669-0)
Supplement: Supplementary file 1 — Additional file 1. Table GRADE-Summary of findings. [file 10195_2022_669_MOESM1_ESM.pdf]

Supplementary material. Table GRADE-Summary of findings

Certainty assessment							Summary of findings				
							Study event rates (%)		Relative effect (95% CI)	Anticipated absolute effects	
Participants (studies) Follow up	Risk of bias	Inconsistency	Indirectness	Imprecision	Publication bias	Overall certainty of evidence	With freehand surgery	With robotic surgery		Risk with freehand surgery	Risk difference with robotic surgery
**Accuracy of pedicle screw placement (Grade A-maximum accuracy) (assessed with: Gertzbein y Robbins scale)**											
3477 (9 RCTs) [13, 18-22, 25-27]	very serious [a]	serious [b]	not serious	not serious	none	⊕◯◯◯ VERY LOW	1515/1779 (85.2%)	1566/1698 (92.2%)	**RR 1.06** (1.01 to 1.11)	852 per 1000	**51 more per 1000** (from 9 more to 94 more)
**Accuracy of pedicle screw placement (Grade A+B-safety zone) (assessed with: Gertzbein y Robbins scale)**											
3477 (9 RCTs) [13, 18-22, 25-27]	very serious [a]	serious [b]	not serious	not serious	none	⊕◯◯◯ VERY LOW	1706/1779 (95.9%)	1665/1698 (98.1%)	**RR 1.06** (1.01 to 1.11)	959 per 1000	**10 more per 1000** (from 0 fewer to 29 more)
**Proximal facet violation**											
1716 (3 RCTs)[13, 18, 22]	very serious [c]	not serious	not serious	not serious	none	⊕⊕◯◯ LOW	26/896 (2.9%)	0/820 (0.0%)	**RR 0.07** (0.01 to 0.40)	29 per 1000	**27 fewer per 1000** (from 29 fewer to 17 fewer)

Certainty assessment							Summary of findings				
**Distance between pedicle screw and facet joint surface (mm)**											
138 (2 RCTs) [13, 22]	very serious [d]	not serious	not serious	not serious [e]	none	⨁⨁◯◯ LOW	71	67	-	The mean distance between pedicle screw and facet joint surface (mm) was **0** mm	MD **1.69 mm more** (1.18 more to 2.2 more)
**Mean screw deviation (mm)**											
274 (1 RCT) [20]	serious [f]	not serious	not serious	not serious	none	⨁⨁⨁◯ MODERATE	136	138	-	The mean mean screw deviation (mm) was **0** mm	MD **2.15 mm fewer** (3.06 fewer to 1.24 fewer)
**Convergence angle (degree)**											
78 (1 RCT) [22]	very serious [g]	not serious	not serious	not serious [e]	none	⨁⨁◯◯ LOW	41	37	-	The mean convergence angle (degree) was **0** mm	MD **6 mm more** (3.45 more to 8.55 more)
**Intraoperative blood loss (mL)**											
394 (3 RCTs) [13, 25, 27]	serious [h]	not serious	not serious	very serious [i]	none	⨁◯◯◯ VERY LOW	199	195	-	The mean intraoperative blood loss (mL) was **0** mL	MD **68.12 mL fewer** (109.24 fewer to 27.01 fewer)

Certainty assessment							Summary of findings				
**Radiation dose (Standard mean difference. Measurements were made in different units: μSv, mSv, and mGy)**											
402 (4 RCTs) [13, 19, 21, 25]	very serious [j]	serious [k]	not serious	not serious	none	⊕◯◯◯ VERY LOW	203	199	-	-	SMD **1.31 SD fewer** (2.02 fewer to 0.6 fewer)
**Fluoroscopic time (sec)**											
262 (2 RCTs) [13, 19]	very serious [l]	not serious	not serious	serious [m]	none	⊕◯◯◯ VERY LOW	129	133	-	The mean fluoroscopic time (sec) was **0** sec	MD **3 sec lower** (28 lower to 22 higher)
**Total screw placement time (min)**											
108 (2 RCTs) [19, 25]	very serious [l]	not serious	not serious	very serious [n]	none	⊕◯◯◯ VERY LOW	50	58	-	The mean total screw placement time (min) was **0** min	MD **0.84 min higher** (10.93 lower to 12.61 higher)
**Operating time (min)**											
492 (5 RCTs) [13, 20-22, 25]	very serious [o]	not serious	not serious	not serious	none	⊕⊕◯◯ LOW	247	245	-	The mean duration of the intervention (min) was **207** minutos	MD **6.45 minutos more** (13.59 fewer to 26.49 more)

Certainty assessment							Summary of findings				
**Length of hospital stay (days)**											
374 (3 RCTs)[13, 21, 25]	serious [p]	serious [q]	not serious	serious [m]	none	⊕◯◯◯ VERY LOW	189	185	-	The mean length of hospital stay (days) was **189**	MD **0.36 lower** (1.03 lower to 0.31 higher)
**Back pain change (Visual Analog Pain Scale)**											
130 (2 RCTs) [13, 22]FENG	very serious [r]	not serious	not serious	not serious	none	⊕⊕◯◯ LOW	67	63	-	The mean back pain change (Visual Analog Pain Scale) was **0**	MD **0.06 higher** (0.53 lower to 0.64 higher)
**Leg pain change (Visual Analog Pain Scale)**											
130 (2 RCTs) [13, 22]FENF	very serious [r]	not serious	not serious	not serious	none	⊕⊕◯◯ LOW	67	63	-	The mean leg pain change (Visual Analog Pain Scale) was **0**	MD **0.13 lower** (0.75 lower to 0.49 higher)
**Oswestry Disability Index**											
130 (2 RCTs) [13, 22]FENG	very serious [r]	not serious	not serious	not serious	none	⊕⊕◯◯ LOW	67	63	-	The mean oswestry Disability Index was **0**	MD **0.58 lower** (4.19 lower to 3.03 higher)

Certainty assessment							Summary of findings				
**Short Form 36-item Health Survey (physical health)**											
70<br>(1 RCT) [22]	very serious [g]	not serious	not serious	not serious	none	⊕⊕◯◯<br>LOW	33	37	-	The mean short Form 36-item Health Survey (physical health) was **0**	MD **5.4 higher**<br>(1.03 lower to 11.83 higher)
**Short Form 36-item Health Survey (mental health)**											
70<br>(1 RCT) [22]	very serious [g]	not serious	not serious	not serious	none	⊕⊕◯◯<br>LOW	33	37	-	The mean short Form 36-item Health Survey (mental health) was **0**	MD **0.7 lower**<br>(9.13 lower to 7.73 higher)
**Surgical Site Infection**											
207<br>(2 RCT) [25, 26]	serious [f]	not serious	not serious	very serious [i]	none	⊕◯◯◯<br>VERY LOW	3/106<br>(2.8%)	1/101<br>(1.0%)	**RR 0.45**<br>(0.07 to 2.98)	28 per 1000	**16 fewer per 1000**<br>(from 26 fewer to 56 more)
**Other complications**											

Certainty assessment							Summary of findings				
207<br>(2 RCT) [25, 26]	serious [f]	not serious	not serious	serious [m]	none	⨁⨁◯◯<br>LOW	7/106<br>(6.6%)	1/101<br>(1.0%)	**RR 0.21**<br>(0.04 to 1.20)	66 per 1000	**52 fewer per 1000**<br>(from 63 fewer to 13 more)

CI: Confidence interval; **RR:** Risk ratio; **MD:** Mean difference; **SMD:** Standardized mean difference

Explanations

a. High risk of bias due to high or unclear risk of random sequence generation or allocation concealment was observed in five of seven studies. Performance risk of bias was observed in five studies and lack of blinding of outcome investigations in two studies. Unclear risk due to incomplete data was observed in two studies and reporting bias in one study.
b. Unexplained heterogeneity; I^2=75% (inconsistency -1)
c. High risk of bias was observed due to allocation concealment and lack of blinding of participant, personnel and investigators in one study and unclear risk in detention bias in two studies.
d. High risk of bias was observed due to allocation concealment and lack of blinding of participant, personnel and investigators in one study and unclear risk in detention bias in other study.
e. Very small sample size and narrow confidence interval not crossing the line of no effect. No imprecision.
f. Unclear bias in random sequence generation, allocation concealment and participants and personnel blinding was observed.
g. High risk related with allocation concealment and lack of participants, personnel blinding was observed. The study shows unclear risk in random sequence generation.
h. Unclear risk of selection bias, detection bias and attrition bias has been observed.
i. Small sample size and very wide confidence interval crossing the line of no effect (imprecision −2)
j. One study shows a high risk of bias in random sequence generation and allocation concealment and lack of participant, personnel and investigators blinding. Others studies present unclear risk in selection bias, detection bias an attrition bias.
k. Unexplained heterogeneity; I^2=83% (inconsistency -1)
l. High or unclear of risk bias due to problems in random sequence generation and allocation concealment and lack of participant, personnel and investigators blinding were observed.
m. Small sample size and wide confidence interval crossing the line of no effect (imprecision −1).
n. Very small sample size and wide confidence interval crossing the line of no effect (imprecision −2).
o. High or unclear of risk bias due to problems in random sequence generation and allocation concealment and lack of participant, personnel and investigators blinding were observed.
p. Unclear bias in related with random sequence generation, allocation concealment, blinding of participants and personnel and unclear detection bias and attrition bias was observed.
q. Unexplained heterogeneity; I^2=62% (inconsistency -1)
r. High risk or unclear bias related with random sequence generation, allocation concealment and lack of participants, personnel and investigators blinding was observed.
